# High-throughput testing in head and neck squamous cell carcinoma identifies agents with preferential activity in human papillomavirus-positive or negative cell lines

**DOI:** 10.18632/oncotarget.25436

**Published:** 2018-05-25

**Authors:** Farhad Ghasemi, Morgan Black, Ren X. Sun, Frederick Vizeacoumar, Nicole Pinto, Kara M. Ruicci, John Yoo, Kevin Fung, Danielle MacNeil, David A. Palma, Eric Winquist, Joe S. Mymryk, Laurie A. Ailles, Alessandro Datti, John W. Barrett, Paul C. Boutros, Anthony C. Nichols

**Affiliations:** ^1^ Department of Otolaryngology – Head and Neck Surgery, London Health Sciences Centre, London, Ontario, Canada; ^2^ Department of Oncology, London Health Sciences Centre, London, Ontario, Canada; ^3^ Ontario Institute of Cancer Research, MaRS Centre, Toronto, Ontario, Canada; ^4^ Department of Pharmacology & Toxicology, University of Toronto, Toronto, Ontario, Canada; ^5^ Cancer Research Cluster, University of Saskatchewan, Saskatoon, Saskatchewan, Canada; ^6^ Department of Microbiology and Immunology, University of Western Ontario, London, Ontario, Canada; ^7^ Department of Medical Biophysics, University of Toronto, Toronto, Ontario, Canada; ^8^ Network Biology Collaborative Centre, Lunenfeld Tanenbaum Research Institute, Mount Sinai Hospital, Toronto, Ontario, Canada; ^9^ Department of Agricultural, Food, and Environmental Sciences, University of Perugia, Perugia, Italy

**Keywords:** head and neck cancer, human papillomavirus, high throughput drug testing, cell lines, chemotherapy

## Abstract

Head and neck squamous cell carcinoma (HNSCC) is a common cancer diagnosis worldwide. Despite advances in treatment, HNSCC has very poor survival outcomes, emphasizing an ongoing need for development of improved therapeutic options. The distinct tumor characteristics of human papillomavirus (HPV)-positive *vs*. HPV-negative disease necessitate development of treatment strategies tailored to tumor HPV-status. High-throughput robotic screening of 1,433 biologically and pharmacologically relevant compounds at a single dose (4 μM) was carried out against 6 HPV-positive and 20 HPV-negative HNSCC cell lines for preliminary identification of therapeutically relevant compounds. Statistical analysis was further carried out to differentiate compounds with preferential activity against cell lines stratified by the HPV-status. These analyses yielded 57 compounds with higher activity in HPV-negative cell lines, and 34 with higher-activity in HPV-positive ones. Multi-point dose-response curves were generated for six of these compounds (Ryuvidine, MK-1775, SNS-032, Flavopiridol, AZD-7762 and ARP-101), confirming Ryuvidine to have preferential potency against HPV-negative cell lines, and MK-1775 to have preferential potency against HPV-positive cell lines. These data comprise a valuable resource for further investigation of compounds with therapeutic potential in the HNSCC.

## INTRODUCTION

There has been an epidemic rise in oropharyngeal cancer worldwide due to increasing rates of oral infection with human papillomavirus (HPV) [1, 2]. HPV-positive cancers typically arise in younger and healthier patients that consume less alcohol and tobacco than patients with HPV-negative head and neck squamous cell cancers (HNSCC). Although HPV-positive patients are much more likely to be cured of their disease [1, 3], an important challenge for these patients has been enduring the acute and chronic toxicities of therapy. In addition, HPV-positive tumors are distinct from a molecular perspective, with characteristic genetic, epigenetic and protein profiles [4]. Given these profound differences between HPV-positive and HPV-negative disease at the clinical and molecular level, it is logical that the development of any therapy is tailored specifically for the tumor HPV-status.

Cell lines are imperfect models of cancer [5, 6], however HNSCC cell lines appear to have a similar genomic landscape to primary tumors [7, 8]. More importantly, genomic markers of drug sensitivity in cell lines appear to generally correlate well with validated biomarkers in patient tumors [7, 9]. Thus, as cell lines can be easily and cheaply screened with large collections of drugs, they are invaluable tools to identify new agents with potent activity against tumor cells.

This study aimed to carry out a high-throughput drug screen of a large collection of compounds against HNSCC cell lines, with the objective of preliminary identification of potent and HPV-status selective agents suitable for further investigation.

## RESULTS

### High-throughput screening identified multiple drugs with high potency against HNSCC cell lines

**Figure 1:**
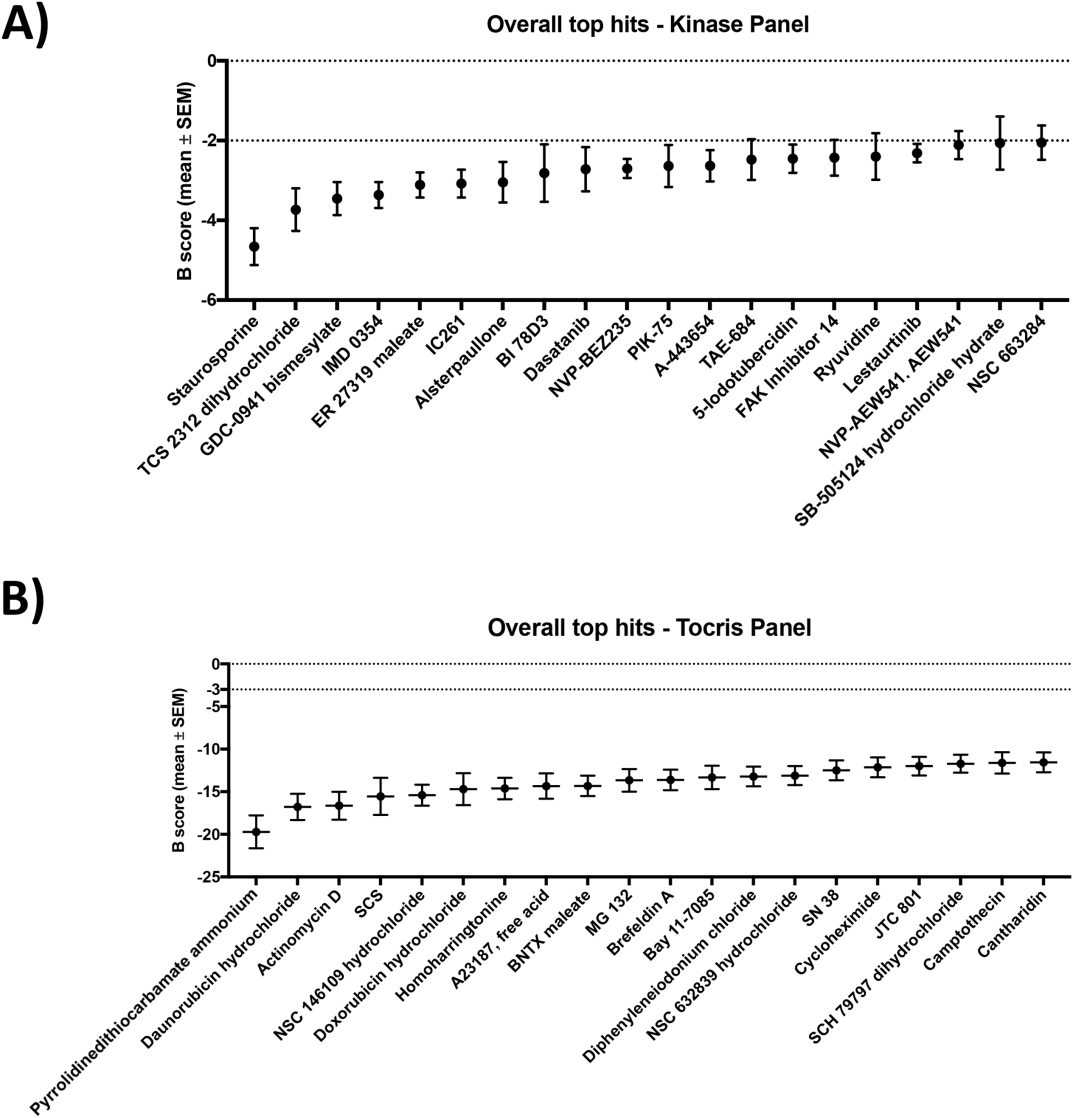
The top 20 most potent drugs and determined by the B-score in HNSCC cell lines from the Kinase inhibitor **(A)** and Tocris **(B)** panels in the high-throughput drug screen.

The top 20 compounds with the highest activity from the kinase inhibitor and Tocris panels are reported in Figure 1A and 1B, respectively (List of compounds and mechanism of action can be found in Supplementary Table 1). This screen identified drugs with high potency that included a diverse range of chemotherapeutic and targeted agents, however several themes emerged (Supplementary Figure 1). Four inhibitors of the phosphoinositide 3 kinase (PI3K) pathway were identified (GDC0941, PIK-75, NVP-BEZ235, FAK Inhibitor 14), as were 3 drugs reported to affect various aspect of the cell cycle (MK 1775, TCS2312, NSC 146109). Three topoisomerase inhibitors were potent (Daunorubicin, Doxorubicin and SN 38). Multiple compounds affecting inflammatory pathways through inhibition of proteins such as NF-κB, IKKβ and IκBα were also identified (IMD0354, Pyrrolidinedithiocarbamate ammonium, MG 132 and Bay 11-7085). Cisplatin and carboplatin, chemotherapeutics in routine use head and neck cancer, did not meet our criteria for significant activity (B-score < −2) in the cell line panel at a dose of 4μM (Supplementary Figure 2). Complete tables of individual compound activity against each cell line from the kinase inhibitor and Tocris panels are provided in Supplementary Table 2.

### A subset of compounds demonstrated preferential activity against cell lines based on HPV-status

**Figure 2:**
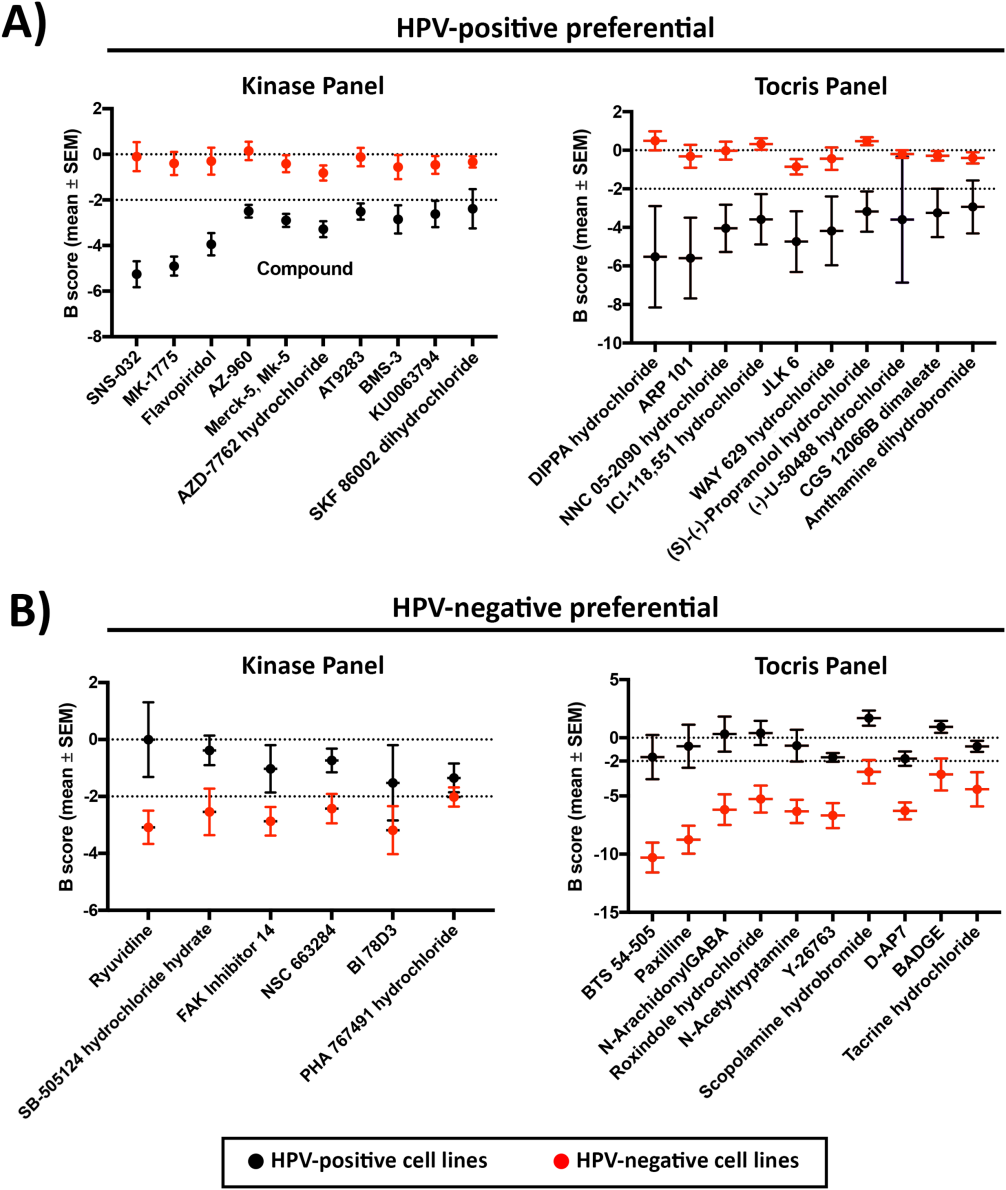
Top 10 drugs with **(A)** HPV-positive and **(B)** HPV-negative selective potency from Kinase inhibitor and Tocris compound panels.

Thirty compounds from the kinase panel, and 27 compounds from the Tocris panel were found to be more potent against HPV-positive cell lines (Figure 2A shows top hits, full list in Supplementary Table 3). Among the identified hits, multiple compounds impacted cell cycle pathways (Supplementary Figure 1), including inhibition of cyclin-dependent kinases (CDK: SNS-032, Alvocidib), PI3K (PI-103, PIK90, Deguelin), polo-like kinase 1 (PLK1: GSK-461364, BI 2536), and checkpoint kinases (CHK: AZD-7762, SB 218078). Various Janus kinase (JAK) inhibitors were also seen in the HPV-positive preferential list (AZ-960, Merck-5, AT9283, Lestaurtinib). The compounds DIPPA hydrochloride, MVC 05-290, Way 629, Physostigmine and PNC 120596 that are known to act on neuro-receptors were demonstrated to be more potent in the HPV-positive cell lines.

Six compounds from the kinase inhibitor panel and 28 compounds from the Tocris panel were found to have higher activity in HPV-negative cell lines (Figure 2B shows top hits, full list in Supplementary Table 4). Three compounds impacting cellular proliferation and differentiation were identified (Supplementary Figure 1), including SB-505124, focal adhesion kinase (FAK) Inhibitor 14 and Aminopurvalanol A. From the Tocris panel, compounds impacting various aspects of cellular regulation and physiology were common hits against HPV-negative cell lines (Supplementary Figure 1), including Y-26763, D-AP7, BADGE, SKF 96365 hydrochloride, Olvanil, Embelin, GR 127935 hydrochloride, Demethylasterriquinone B1, Tyrphostin B44, Ro 90-7501, PALDA and OLDA.

### Dose response curves confirm Ryuvidine and MK-1775 to be preferentially potent against HPV-negative and positive cell lines respectively

**Figure 3:**
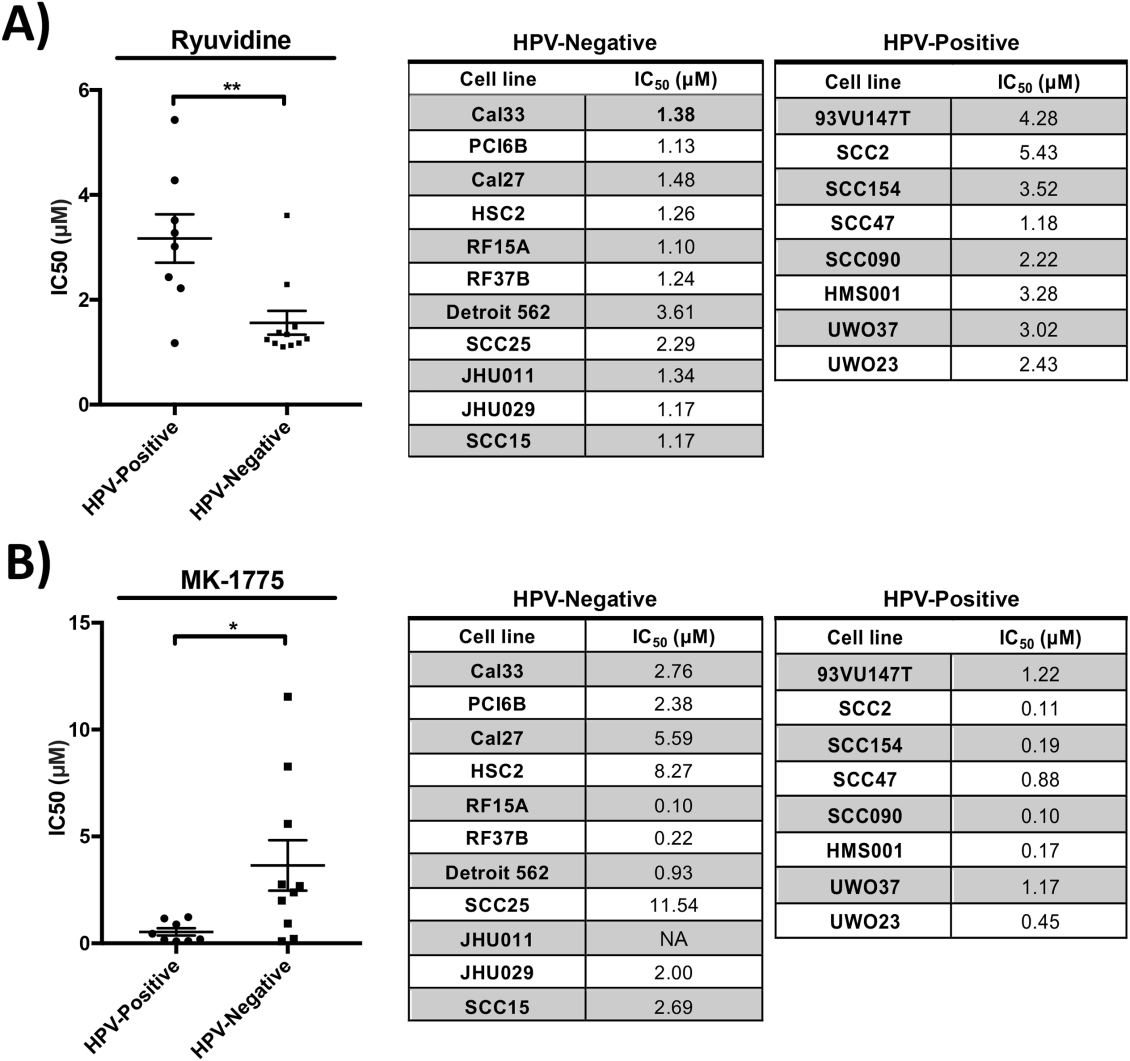
Drug potency validation studies revealed **(A)** Ryuvidine to be selective against HPV-negative cell lines (^**^ p=0.003) and **(B)** MK-1775 to be selective against HPV-positive cell lines (^*^ p=0.03).

After the generation of multi-point dose response curves, Ryuvidine was selectively potent against HPV-negative cell lines, with an average IC_50_ of 1.562 μM ± 0.755 (mean ± standard deviation) in HPV-negative and 3.169 μM ± 1.305 in HPV-positive cell lines (Figure 3A, unpaired *t*-test p=0.003), confirming the findings in the high-throughput screen. MK-1775 showed significantly higher potency against HPV-positive cell lines with an average IC_50_ of 0.535 μM ± 0.4828 in HPV-positive and 3.647 μM ± 3.73 in HPV-negative cell lines (Figure 3B, unpaired *t*-test p=0.03), again showing compatible findings with the high-throughput drug screen. SNS-032, Flavopiridol, AZD-7762 and ARP-101 compounds did not show significantly different potency in cell lines stratified by HPV-status following validation studies across a range of concentrations (Supplementary Figure 3, unpaired *t*-test p>0.05). Dose-response studies of MK-1775, Ryuvidine, AZD7762 and Flavopiridol in control non-cancerous IMR90 cell line showed higher IC_50_ values (IC_50_ of >10 μM, 2.2 μM, 0.61 μM and >10 μM, respectively) compared to susceptible HNSCC cell lines, demonstrating the presence of a therapeutic window.

## DISCUSSION

To our knowledge, this is the first high-throughput drug screening study that focuses specifically on head and neck cancer cell lines. Our study of 1,433 compounds in 26 cell lines identified 57 and 34 compounds with preferential activity against HPV-negative and HPV-positive cell lines, respectively. Validation studies with six of these confirmed selective potencies in HPV-negative (Ryuvidine) and HPV-positive (MK-1775) cell lines. Given that HPV-positive and negative tumors are distinct from a molecular and prognostic perspective [10], this first step in the development of therapeutics specific to each disease type is critical to achieve the end goal of maximizing survival rates while minimizing toxicity.

Multiple studies and clinical trials have identified a superior response to chemotherapy, and better survival outcomes in HPV-positive HNSCCs compared to the HPV-negative tumors [3, 11–14]. Following these observations, treatment plans and clinical trials for HNSCC are increasingly being tailored based on tumor HPV status, particularly with the aim to de-intensify treatment for patients with HPV-positive tumors [15]. It is notable that despite the overall favourable outcomes in HPV-positive tumors, treatment failure still occurs in 20-40% of advanced disease [16]. As a result, there remains a necessity for further discovery and development of selective therapeutics in both HPV-positive and negative cohorts of HNSCCs.

Ryuvidine functions as an inhibitor of the CDK4 and SETD8 proteins [17, 18]. CDK4, and its close relative CDK6, interact with cyclin D and function in a pathway that is important for cell cycle progression. The CDK4/cyclin D complex impacts cell cycle progression by phosphorylating and inactivating retinoblastoma (Rb), a negative regulator of G1 exit [19]. Interestingly, Cyclin D1 is amplified frequently in HPV-negative HNSCC and p16, the negative regulator of the kinase complex, is frequently deleted [20]. Thus, Ryuvidine may reverse the effects of these mutations, leading to cell cycle arrest in HPV-negative HNSCC. In contrast, the Rb pathway is abrogated in HPV-positive cells as the HPV E7 oncoprotein binds Rb and targets it for degradation [21]. Thus, the drug would not be expected to have an effect in HPV-positive cells, which is consistent with our finding (Figure 3A). Further investigation will be required to clarify the mechanism behind the observed preferential potency of Ryuvidine against HPV-negative cell lines.

MK-1775, also known as AZD1775, is a WEE1 kinase inhibitor [22]. WEE1 kinase has been linked to G2-M cell cycle arrest in response to DNA damage. Inhibition of WEE1 kinase can therefore overcome G2 cell cycle arrest, which in the presence of DNA damage, can lead to mitotic catastrophe and ultimately cell death [22, 23]. As a result of this mechanism of action, WEE1 inhibitors have entered clinical trials as chemo-sensitizers of various tumors, such as breast cancer, leukemia and melanoma [24]. Previous studies have shown that MK-1775 can sensitize both HPV-positive and HPV-negative cell lines to cisplatin therapy, but through differing mechanisms; senescence [25] versus apoptosis [26] in HPV-negative and HPV-positive cell lines, respectively. Our validation study revealed MK-1775 to be independently potent in reducing cellular proliferation, with preferential activity in HPV-positive cell lines (Figure 3B). WEE1 kinase inhibition has been shown to selectively sensitize p53-deficient cells to exogenous DNA damage [25], and the higher susceptibility of HPV-positive cell lines to MK1775 may be due to the inhibition of p53 by the HPV E6 protein [27]. Further studies may be indicated to explore the potential of MK1775 compound as an HPV-specific chemotherapeutic agent.

Preliminary dose-response curve validation studies were able to validate the selectivity of 2 of the 6 tested compounds that were derived based on our high-throughput study. This is not surprising as the high-throughput study included only a single dose of each compound, with the purpose of preliminary identification of compounds with potential for further assessment. More of the identified compounds may show HPV-status specific activity against HNSCCs following validation. Furthermore, analyses of the available HNSCC cell lines have revealed common genetic alterations other than HPV-status that are also reflected in tumor populations [28]. These include amplifications in PIK3CA, EGFR and CCND2, deletions in CDKN2A, SMAD4 and NOTCH2, and mutations in TP53, CDKN2A, SMAD4 and PIK3CA [28]. Thus, the genetic characterization of our cell line panel paired with our high-throughput drug screen can potentially be used to identify genomic correlates of drug response as a further step towards providing personalize medicine for head and neck cancer patients.

High-throughput testing of large chemical compound libraries can serve as valuable resources in the context of a necessity for better chemotherapeutics in the field of head and neck oncology. In this study, we have provided the head and neck cancer research community with the results of a preliminary screen of the activity of over 1400 compounds against 26 HNSCC cell lines. Initial validation studies led to the identification of Ryuvidine and MK-1775 compounds to have preferential activity against HPV-negative and HPV-positive cell lines respectively. We hope that the provided datasets can serve as a valuable resource to lay the foundation for further investigation of compounds with therapeutic potential in the field of head and neck oncology.

## MATERIALS AND METHODS

### Cell lines and culture conditions

Twenty-eight HNSCC cell lines were obtained from established culture collections or from collaborators as listed in Supplementary Table 5. Short tandem repeat profiling was carried out as previously described [29] and compared to the literature to confirm the cell line identity (Supplementary Table 6). Cells were cultured in an incubator at 37°C with 5% CO_2_ in the medium specified in Supplementary Table 5.

### High-throughput drug screening

A custom-made compound library from Tocris Bioscience comprised of 1,113 compounds and a kinase inhibitor library of 320 compounds were used for high throughput drug screening. The drug screen was carried out against 6 HPV-positive and 20 HPV-negative cell lines (Supplementary Table 5). High-throughput drug studies were carried out in the S.M.A.R.T Facility in the Samuel Lunenfeld Research Institute at Mount Sinai Hospital in Toronto, Ontario, Canada. Cells were robotically seeded at a density of 600 cells/well in 384-well plates and incubated for 24 hours prior to the addition of drugs using an automated drug pinning device (Beckman Multimek, Mississauga, Ontario). Cells were treated with either DMSO (control) or a single dose of each drug at a concentration of 4 μM, and cells were subsequently incubated for 48 hours based on methodology used in previous high-throughput studies [30, 31]. Cell viability was measured indirectly using the AlamarBlue reagent (ThermoFisher Scientific).

### Statistical analysis

Fluorescence intensity derived from the high-throughput screen were normalized using B scores as per recommendation of Malo *et. al.* [32]. For cell lines with two replicates, the average B score per drug was calculated. In each of the drug panels, the most potent drugs were identified as those with an average B score below −2. Drugs were then sorted based on the highest B score to the lowest within that stratification scheme. To identify drugs with preferential activity based on HPV-status, Student's *t*-tests were utilized to compare the average B score of HPV-positive cell lines to that of HPV-negative cell lines for each drug. These p-values were then controlled for local false discovery rates using the *fdrtool* package version 1.2.15 [33] in the R statistical environment (version 3.4.0). The statistical comparison was considered significant if the corrected p-value after correction by local false discovery rate (LFDR) was lower than 0.05. Drugs with significant preferential activity were then isolated by having a B score of lower than −2 in one disease type (*ie* HPV+ or HPV-) and a B score of higher than −2 in the opposite disease type, then sorted by the mean difference in the two arms of comparison (from high to low).

### Generation of dose response curves

Six drugs (Ryuvidine, MK-1775, SNS-032, Flavopiridol, AZD-7762 and ARP-101) that demonstrated high differential activity in either HPV-positive or negative cell lines and mechanisms of action that were deemed to be interesting in HNSCC were selected for further analysis. Dose response curves were generated, and drug validation studies for these compounds were tested in 8 HPV-positive and 11 HPV negative cell lines (cell lines listed in Supplementary Table 5). Cells were seeded in 96-well plates at 3000 cells/well. After 24 hours compounds were added in a concentration series of 0.01, 0.03, 0.1, 0.3, 1, 3, 10, 30 and 100 μM. Three technical replicates were carried out in each cell line, per drug. Cells were incubated with the added drugs for 72 hours before assessing potency. Cell viability was measured indirectly using the PrestoBlue® Reagent (Thermofisher Scientific). To calculate the half-maximal inhibitory concentration (IC_50_) value for each cell line, normalized Relative Fluorescence Units (RFUs) of the drug-treated replicates were calculated as a percentage of the mean RFU of the vehicle control (DMSO-only) treatment replicates. IC_50_ values, defined as the concentration at which the normalized RFU reached 50%, were calculated by non-linear regression (Prism® 7 Graphpad Software, Inc). When a non-linear regression model could not be converged for a compound and cell line, no IC_50_ value was assigned and data was excluded from comparison analysis. An unpaired *t*-test was used to assess the significance of the differences in IC_50_ values between HPV-positive and HPV-negative cell lines for each of the compounds, and a p value of less than 0.05 was set as the significance limit. As a preliminary assessment of compound toxicity, we also tested 4 of the compounds (MK1775, Ryuvidine, AZD7762 and Flavopiridol) against the IMR90 non-cancerous cell line (ATCC CCL-186) over a dose range of 0.03 μM to 10 μM with the same methodology to generate dose-response curves and calculate IC_50_ values.

## SUPPLEMENTARY MATERIALS FIGURES AND TABLES





## Abbreviations

HNSCC: Head and Neck Squamous Cell Carcinoma
HPV: Human papillomavirus
IC_50_: half-maximal inhibitory concentration
