# Supplementary material for: High-throughput testing in head and neck squamous cell carcinoma identifies agents with preferential activity in human papillomavirus-positive or negative cell lines
Source: Oncotarget. 2018 May 25;9(40):26064–71. doi: 10.18632/oncotarget.25436 (PMC5995257; doi:10.18632/oncotarget.25436)
Supplement: Supplementary file 1 [file oncotarget-09-26064-s001.pdf]

High-throughput testing in head and neck squamous cell carcinoma identifies agents with preferential activity in human papillomavirus-positive or negative cell lines

SUPPLEMENTARY MATERIALS

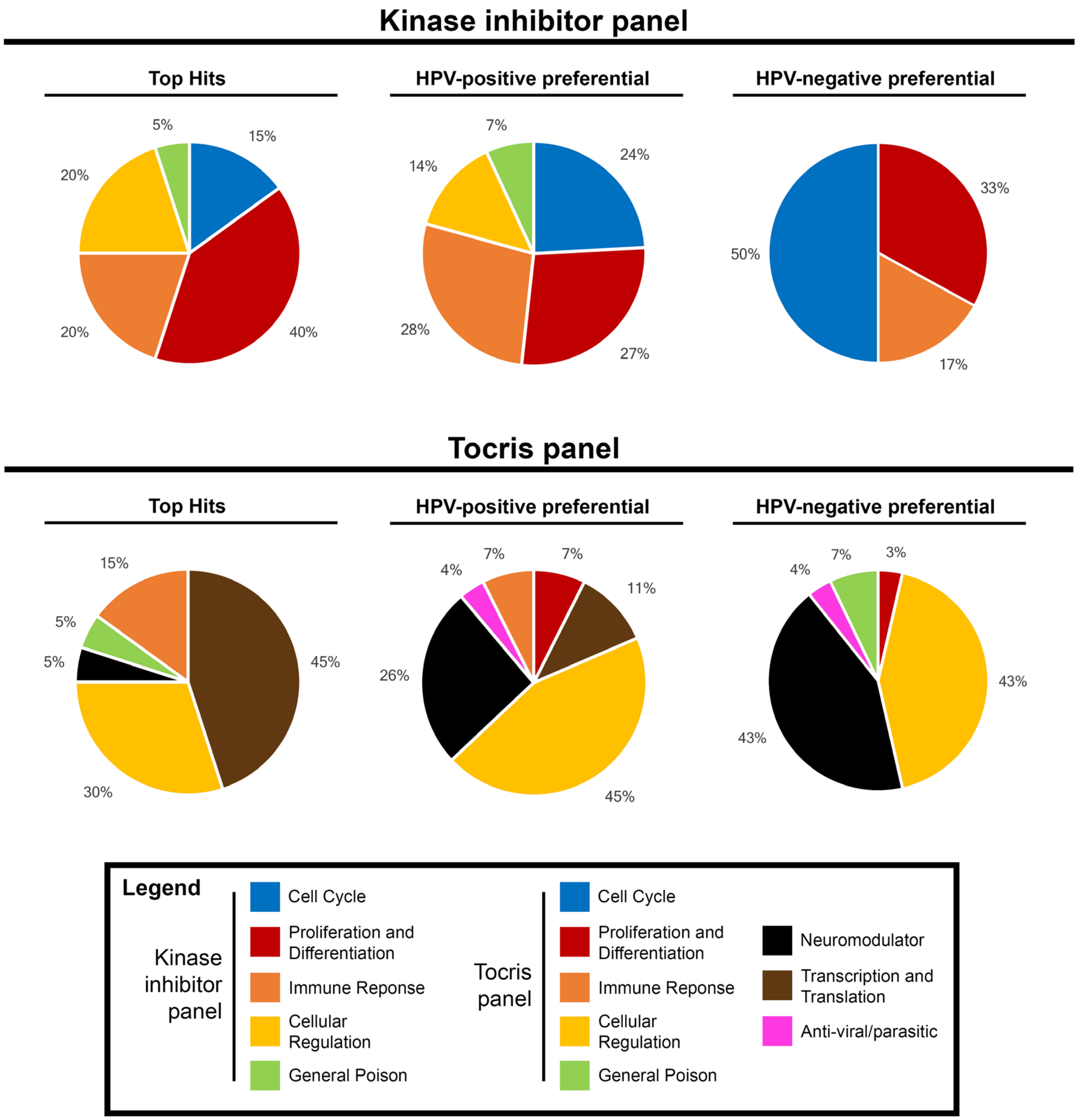

Supplementary Figure 1: Compounds identified as top hits in potency, preferential activity against HPV-positive and preferential activity against HPV-negative cell lines using the Kinase inhibitor and Tocris panels grouped by their mechanism of action.

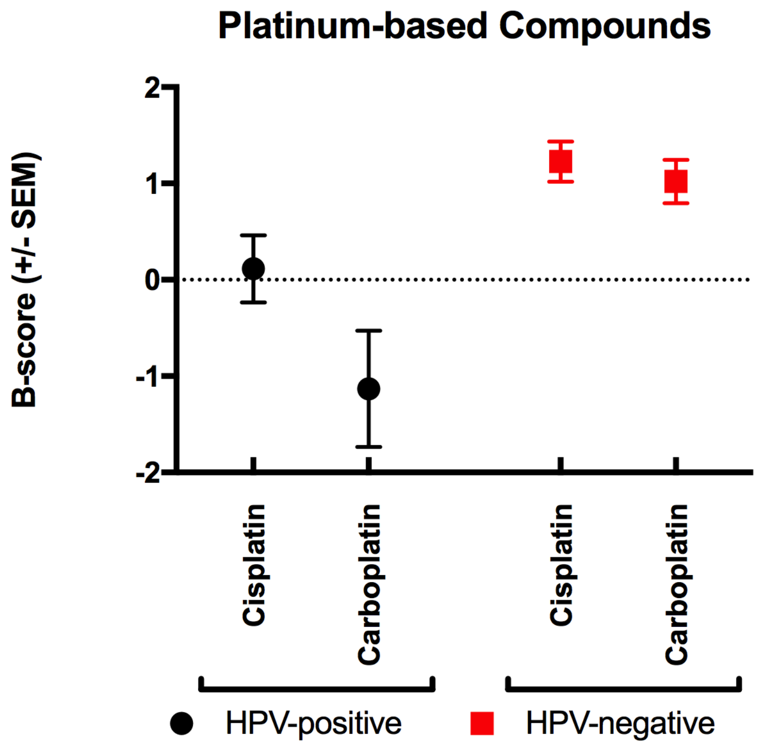

Supplementary Figure 2: Cisplatin and carboplatin, chemotherapeutics in routine use head and neck cancer, were included in the compound panels, but did not meet the set criteria for significant activity (B-score < -2) at a dose of 4µM.

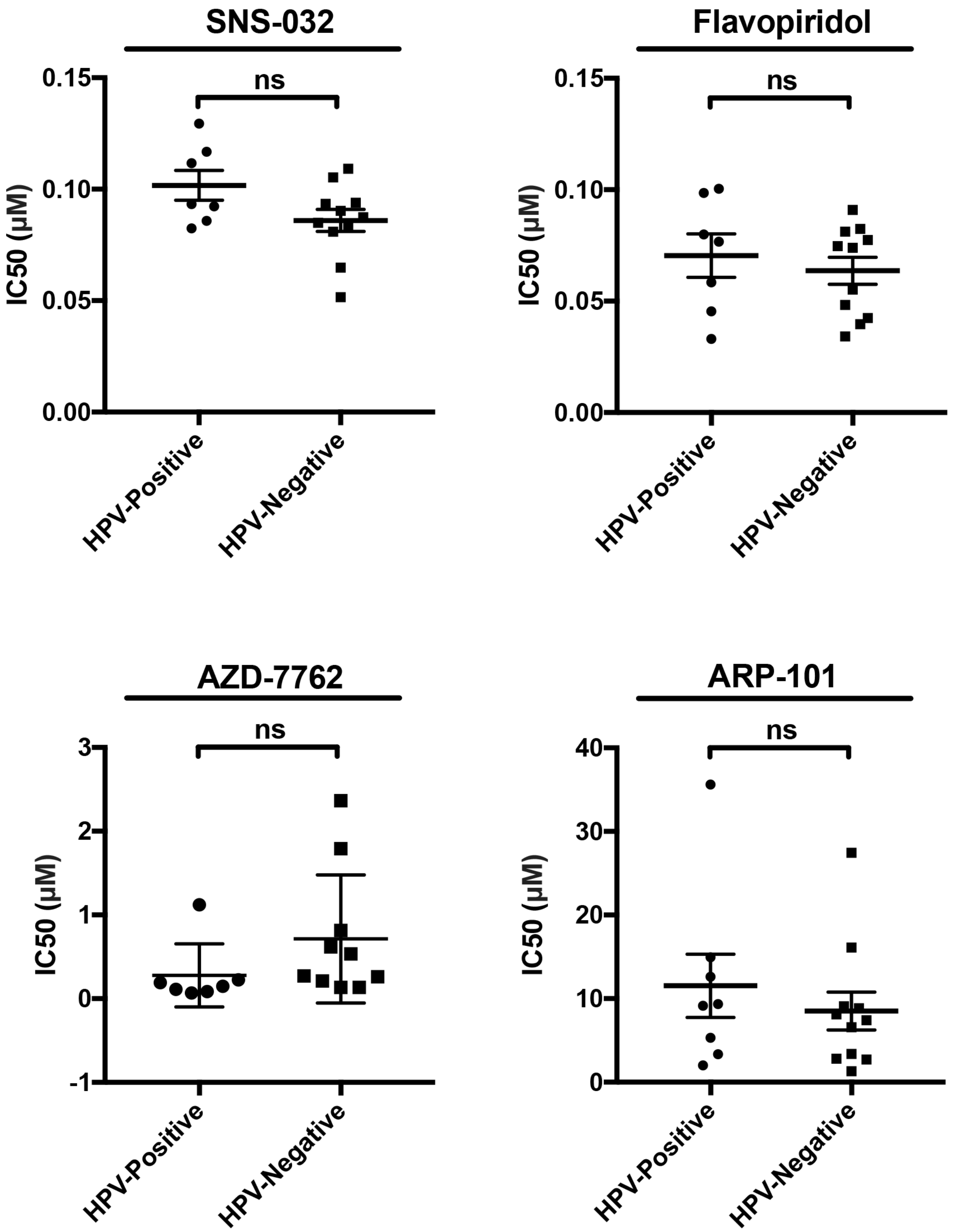

Supplementary Figure 3: Validation studies on SNS-032, Flavopiridol, AZD-7762 and ARP-101 did not reveal significantly selective potency against HNSCC cell lines stratified by HPV status (p=0.07, 0.54, 0.19 and 0.48 respectively). (ns = not significant).

Supplementary Table 1: Top 20 compounds with highest potency from the kinase inhibitor and tocris panels against HNSCC cell lines

Compound	All Cell lines		Primary mechanism of action	Category
	Mean	SEM		
**Top 20 overall Hits from Kinase Inhibitor Panel**				
**Staurosporine**	-4.66	0.46	Non-selective protein kinase inhibitor	General Poison
**TCS 2312 dihydrochloride**	-3.73	0.54	Potent Chk1 inhibitor	Cell Cycle
**GDC-0941 bismesylate**	-3.45	0.41	Inhibitor of class I PI3 kinase	Proliferation & Differentiation
**IMD 0354**	-3.36	0.33	Inhibitor of IKKβ	Immune Response
**ER 27319 maleate**	-3.11	0.31	Selective Syk kinase inhibitor	Immune Response
**IC261**	-3.08	0.35	Selective casein kinase 1δ and 1ε inhibitor	Cellular Regulation
**Alsterpaullone**	-3.04	0.51	Inhibitor of multiple CDKs	Cell Cycle
**BI 78D3**	-2.81	0.72	Competitive JNK inhibitor	Immune Response
**Dasatanib**	-2.72	0.56	Inhibitor of Abl, Src and c-Kit	Proliferation & Differentiation
**NVP-BEZ235**	-2.70	0.24	Inhibitor of PI3K and mTOR	Proliferation & Differentiation
**PIK-75**	-2.64	0.52	Inhibitor of p110α	Proliferation & Differentiation
**A-443654**	-2.63	0.39	Inhibitor of Akt	Proliferation & Differentiation
**TAE-684**	-2.48	0.51	Inhibitor of ALK	Proliferation & Differentiation
**5-Iodotubercidin**	-2.45	0.35	Potent adenosine kinase inhibitor	Cellular Regulation
**FAK Inhibitor 14**	-2.43	0.45	Selective FAK inhibitor	Proliferation & Differentiation
**Ryuvidine**	-2.40	0.58	Inhibitor of SETD8 and CDK4	Cellular Regulation
**Lestaurtinib**	-2.31	0.23	Inhibitor of JAK2, FLT3 and TrkA	Immune Response
**NVP-AEW541, AEW541**	-2.11	0.35	Inhibitor of IGF-1R/InsR	Cellular Regulation
**SB-505124 hydrochloride hydrate**	-2.06	0.67	Inhibitor of ALK5	Proliferation & Differentiation
**NSC 663284**	-2.05	0.43	Inhibitor of Cdc25 phosphatase	Cell Cycle
**Top 20 Overall Hits from Tocris panel**				
**Pyrrolidinedithiocarbamate ammonium**	-19.71	1.92	Inhibits NF-κB, prevents increase in NOS mRNA	Immune Response
**Daunorubicin hydrochloride**	-16.78	1.53	Inhibits topoisomerase II	Transcription and translation
**Actinomycin D**	-16.64	1.64	Inhibits RNA polymerase	Transcription and translation
**SCS**	-15.54	2.18	Selective GABAA receptor antagonist	Neuromodulators
**NSC 146109 hydrochloride**	-15.41	1.23	activates p53-dependent transcription	Transcription and translation
**Doxorubicin hydrochloride**	-14.70	1.88	Inhibits DNA topoisomerase II	Transcription and translation
**Homoharringtonine**	-14.62	1.26	Inhibitor of protein synthesis	Transcription and translation
**A23187, free acid**	-14.34	1.49	Calcium ionophore	General Poison
**BNTX maleate**	-14.31	1.20	Standard δ1 selective antagonist	Cellular regulation & physiology
**MG 132**	-13.67	1.32	Inhibitor of Proteasome, calpain and NF-κB activation	Immune Response
**Brefeldin A**	-13.61	1.21	Disrupts protein translocation to Golgi	Transcription and translation
**Bay 11-7085**	-13.32	1.38	Inhibitor of TNF-α-induced IκBα phosphorylation	Immune Response
**Diphenyleneiodonium chloride**	-13.21	1.15	GPR3 agonist; also inhibits NOS and NADPH oxidases	Cellular regulation & physiology
**NSC 632839 hydrochloride**	-13.11	1.11	Inhibitor of ubiquitin isopeptidase activity	Cellular regulation & physiology
**SN 38**	-12.50	1.17	Inhibitor of DNA topoisomerase I	Transcription and translation
**Cycloheximide**	-12.14	1.16	Inhibitor of protein synthesis	Transcription and translation
**JTC 801**	-12.00	1.10	Selective NOP antagonist	Cellular regulation & physiology
**SCH 79797 dihydrochloride**	-11.72	1.05	Selective casein kinase 1δ and 1ε inhibitor	Cellular regulation & physiology
**Camptothecin**	-11.61	1.25	DNA topoisomerase inhibitor	Transcription and translation
**Cantharidin**	-11.56	1.17	Inhibitor of Protein phosphatase 1/2A	Cellular regulation & physiology

Supplementary Table 2: Complete list of kinase inhibitor panel and Tocris panel compound activity against individual HNSCC cell lines

See Supplementary File 1

Supplementary Table 3: Compounds with preferential activity against HPV-positive cell lines

Compound	HPV-Positive		HPV-Negative		Difference		Primary mechanism of action	Category
	Mean B score	SEM	Mean B score	SEM	Mean B score	SEM		
**Kinase inhibitor Panel**								
SNS-032, BMS387032	-5.26	0.57	-0.10	0.64	-5.16	0.29	CDK 2 inhibitor	Cell Cycle
MK-1775	-4.90	0.42	-0.40	0.51	-4.50	0.25	Wee1 inhibitor	Cell Cycle
Flavopiridol	-3.94	0.49	-0.30	0.59	-3.64	0.27	CDK9 Kinase Inhibitor	Cell Cycle
AZ-960	-2.50	0.28	0.15	0.40	-2.65	0.18	JAK2 inhibitor	Immune Response
Merck-5, Mk-5	-2.90	0.29	-0.41	0.37	-2.49	0.17	JAK kinase family inhibitor	Immune Response
AZD-7762 hydrochloride	-3.29	0.35	-0.82	0.33	-2.47	0.15	Chk1/2 inhibitor	Cell Cycle
AT9283	-2.51	0.35	-0.12	0.40	-2.39	0.18	JAK2/3 inhibitor	Immune Response
BMS-3	-2.85	0.62	-0.56	0.53	-2.29	0.25	LIMK inhibitor	Cellular Regulation
KU0063794	-2.62	0.58	-0.46	0.39	-2.16	0.20	mTOR inhibitor	Proliferation & Differentiation
SKF 86002 dihydrochloride	-2.38	0.86	-0.33	0.25	-2.05	0.21	p38 MAP kinase inhibitor	Immune Response
RDEA-119, AR-119	-2.98	0.89	-1.16	0.30	-1.82	0.23	MEK1/2 inhibitor	Immune Response
GSK461364	-2.02	0.77	-0.24	0.27	-1.78	0.19	PLK1 inhibitor	Cell Cycle
GSK690693	-2.19	0.41	-0.53	0.21	-1.66	0.12	Akt 1/2/3 inhibitor	Proliferation & Differentiation
Foretinib	-2.28	0.79	-0.65	0.48	-1.63	0.26	HGF receptor family tyrosine kinase inhibtor	Proliferation & Differentiation
TWS119	-2.67	0.44	-1.14	0.42	-1.53	0.20	GSK-3β inhibitor	Cellular Regulation
PD 180970	-2.43	0.17	-0.96	0.46	-1.47	0.19	p210Bcr/Abl kinase inhibitor	Proliferation & Differentiation
BI 2536	-3.00	0.98	-1.56	0.20	-1.43	0.22	PLK1 inhibitor	Cell Cycle
SP-600125	-2.02	0.51	-0.64	0.37	-1.38	0.19	Selective JNK inhibitor	Immune Response
PI-103	-3.01	0.22	-1.73	0.34	-1.28	0.15	multi-targeted PI3K inhibitor for p110α/β/δ/γ	Proliferation & Differentiation
HDS 029	-2.38	0.37	-1.15	0.40	-1.23	0.18	ErbB receptor family inhibitor	Cellular Regulation
Akt 1/2 Kinase inhibitor	-2.73	0.28	-1.54	0.28	-1.19	0.13	Akt 1/2 Kinase Inhibitor	Proliferation & Differentiation
ZM 306416 hydrochloride	-2.17	0.42	-1.01	0.38	-1.15	0.18	VEGF receptor tyrosine kinase inhibitor	Proliferation & Differentiation
SB 218078	-2.77	0.35	-1.65	0.28	-1.12	0.14	Chk1 inhibitor	Cell Cycle
TCS JNK 5a	-2.74	0.30	-1.74	0.59	-1.00	0.25	JNK 2/3 inhibitor	Immune Response
Lestaurtinib	-3.03	0.40	-2.11	0.26	-0.92	0.14	JAK2, FLT3 and TrkA inhibitor	Immune Response
JNJ-10198409	-2.09	0.66	-1.22	0.59	-0.87	0.28	PDGF-RTK inhibitor	Proliferation & Differentiation
PKC-412	-2.50	0.30	-1.72	0.21	-0.78	0.11	Multi-targeted kinase inhibitor	General Poison
EKI-785, CL-387,785	-2.39	0.55	-1.63	0.46	-0.76	0.22	EGFR inhibitor	Cellular Regulation
K-252a	-2.58	0.47	-1.84	0.24	-0.74	0.15	Multi-targeted kinase inhibitor	General Poison
PIK 90	-2.40	0.35	-1.82	0.30	-0.58	0.15	PI3Kα/γ/δ inhibitor	Proliferation & Differentiation
**Tocris Panel**								
DIPPA hydrochloride	-5.52	2.63	0.49	0.50	-6.02	0.58	Selective irreversible κ antagonist	Neuromodulators
ARP 101	-5.59	2.10	-0.31	0.60	-5.28	0.50	Inhibitor of MMP-2	Cellular regulation & physiology
NNC 05-2090	-4.05	1.22	-0.02	0.46	-4.03	0.32	GABA uptake inhibitor	Neuromodulators
ICI-118,551	-3.58	1.30	0.33	0.30	-3.91	0.30	β2 antagonist/inverse agonist	Cellular regulation & physiology
JLK 6	-4.74	1.58	-0.85	0.40	-3.89	0.37	Inhibitor of γ-secretase-mediated βAPP processing	Cellular regulation & physiology
WAY 629 hydrochloride	-4.18	1.78	-0.43	0.58	-3.75	0.44	Selective 5-HT2C agonist	Neuromodulators
(S)-(-)-Propranolol hydrochloride	-3.18	1.05	0.47	0.21	-3.65	0.23	β antagonist	Cellular regulation & physiology
(-)-U-50488 hydrochloride	-3.60	3.26	-0.19	0.20	-3.41	0.68	Standard selective κ agonist	Neuromodulators
CGS 12066B dimaleate	-3.25	1.26	-0.29	0.25	-2.96	0.28	5-HT1B agonist	Cellular regulation & physiology
Amthamine dihydrobromide	-2.94	1.38	-0.39	0.29	-2.55	0.31	Highly selective standard H2 agonist	Immune Response
Evans Blue tetrasodium salt	-2.29	1.09	0.24	0.15	-2.53	0.23	inhibitor of L-glutamate uptake	Cellular regulation & physiology
Naltrindole hydrochloride	-2.86	1.32	-0.34	0.47	-2.52	0.34	Selective non-peptide δ antagonist	Cellular regulation & physiology
Physostigmine hemisulfate	-3.35	0.37	-0.84	0.35	-2.52	0.17	Cholinesterase inhibitor	Neuromodulators
Fenretinide	-2.22	1.19	0.15	0.58	-2.37	0.34	Synthetic retinoid and antiproliferative	Proliferation and differentiation
Cordycepin	-2.36	0.34	-0.18	0.26	-2.18	0.13	RNA synthesis inhibitor	Transcription and translation
PHTPP	-2.67	1.02	-1.01	0.58	-1.66	0.32	Selective ERβ antagonist	Cellular regulation & physiology
Deguelin	-2.02	1.09	-0.50	0.34	-1.52	0.27	Multi-target inhibitor including PI3K	Proliferation and differentiation
GF 109203X	-2.54	0.48	-1.10	0.49	-1.44	0.23	Protein kinase C inhibitor	Cellular regulation & physiology
Acyclovir	-2.03	0.72	-0.65	0.42	-1.38	0.23	Inhibits viral DNA polymerase	Anti-viral/parasitic
PNU 120596	-2.34	0.75	-1.05	0.27	-1.29	0.19	Positive allosteric modulator of α7 nAChR	Neuromodulators
N-Acetyl-N-acetoxy-4-chlorobenzenesulfonamide	-2.71	1.29	-1.54	0.49	-1.17	0.34	Inhibitor of aldehyde dehydrogenase	Cellular regulation & physiology
RITA	-2.40	1.67	-1.24	0.52	-1.16	0.41	MDM2-p53 interaction inhibitor	Transcription and translation
NNC 55-0396 dihydrochloride	-2.31	0.76	-1.18	0.70	-1.13	0.33	Inhibitor of T-type calcium channels	Neuromodulators
Caffeic acid phenethyl ester	-2.40	0.74	-1.39	0.45	-1.01	0.24	Specific inhibitor of NF-κB activation	Immune Response
CD 1530	-2.05	0.40	-1.17	0.64	-0.88	0.28	Potent and selective RARγ agonist	Transcription and translation
BMS 191011	-2.76	0.61	-1.89	0.42	-0.86	0.21	Potent BKCa channel opener	Cellular regulation & physiology
Ceramide	-2.21	0.58	-1.47	0.56	-0.74	0.26	Ser/Thr protein phosphatase activator	Cellular regulation & physiology

Supplementary Table 4: Compounds with preferential activity against HPV-negative cell lines

Compound	HPV-Positive		HPV-Negative		Difference		Primary mechanism of action	Category
	Mean B score	SEM	Mean B score	SEM	Mean B score	SEM		
**Kinase inhibitor Panel**								
Ryuvidine	-0.003	1.31	-3.08	0.58	3.08	0.36	SETD8 and CDK4 inhibitor	Cell Cycle
SB-505124 hydrochloride hydrate	-0.38	0.52	-2.54	0.82	2.16	0.36	ALK 4/5/7 inhibitor	Proliferation & Differentiation
FAK Inhibitor 14	-1.03	0.83	-2.87	0.50	1.84	0.28	FAK inhibitor	Proliferation & Differentiation
NSC 663284	-0.73	0.42	-2.43	0.52	1.69	0.23	CDC 25 phosphatase inhibitor	Cell Cycle
BI 78D3	-1.52	1.32	-3.18	0.84	1.66	0.44	JNK inhibitor	Immune Response
PHA 767491 hydrochloride	-1.35	0.51	-2.02	0.34	0.67	0.18	Cdc7/CDK9	Cell Cycle
**Tocris Panel**								
BTS 54-505	-1.66	1.90	-10.29	1.28	8.63	0.66	Potent serotonin and noradrenalin reuptake inhibitor	Neuromodulators
Paxilline	-0.73	1.85	-8.76	1.20	8.03	0.63	Potassium channel blocker; cellular toxin	General Poison
N-ArachidonylGABA	0.32	1.51	-6.17	1.32	6.49	0.63	Various cell signalling pathways	Neuromodulators
Roxindole hydrochloride	0.41	1.04	-5.27	1.15	5.68	0.52	Dopamine D2 autoreceptor agonist	Neuromodulators
N-Acetyltryptamine	-0.68	1.36	-6.33	1.00	5.65	0.50	Mixed agonist-antagonist at melatonin receptors	Neuromodulators
Y-26763	-1.68	0.38	-6.68	1.07	5.00	0.45	KATP channel openner	Cellular regulation & physiology
Scopolamine hydrobromide	1.69	0.65	-2.92	1.02	4.61	0.44	Muscarinic receptor antagonist	Neuromodulators
D-AP7	-1.79	0.62	-6.28	0.73	4.49	0.33	Specific NMDA antagonist	Cellular regulation & physiology
BADGE	0.94	0.52	-3.15	1.38	4.09	0.58	PPARγ antagonist	Cellular regulation & physiology
Tacrine hydrochloride	-0.74	0.48	-4.43	1.47	3.69	0.62	Cholinesterase inhibitor	Neuromodulators
SKF 96365 hydrochloride	-1.67	1.52	-5.19	0.91	3.52	0.49	TRPC channel blocker	Cellular regulation & physiology
Olvanil	-0.57	0.70	-3.47	0.37	2.90	0.21	Vanilloid receptor agonist	Cellular regulation & physiology
(R)-(+)-HA-966	-0.46	0.89	-3.31	0.51	2.85	0.28	NMDA partial agonist/ antagonist	Neuromodulators
(S)-SNAP 5114	0.63	0.76	-2.14	0.29	2.76	0.20	GABA uptake inhibitor	Neuromodulators
2-Methyl-5-hydroxytryptamine hydrochloride	-0.52	0.54	-3.02	0.53	2.51	0.25	5-HT3 agonist/potent 5-HT6 ligand	Neuromodulators
5,7-Dichlorokynurenic acid	-0.34	0.74	-2.84	0.40	2.50	0.23	Potent NMDA antagonist	Neuromodulators
Embelin	0.11	1.32	-2.37	0.68	2.48	0.39	Inhibitor of X-linked inhibitor of apoptosis (XIAP)	Cellular regulation & physiology
Ivermectin	-0.98	0.89	-3.25	0.82	2.27	0.39	Allosteric modulator of α7 nicotinic receptors	Anti-viral/parasitic
Tomoxetine hydrochloride	-0.22	0.35	-2.32	1.14	2.11	0.48	Selective noradrenalin re-uptake inhibitor	Neuromodulators
GR 127935 hydrochloride	-0.29	1.02	-2.38	0.75	2.09	0.38	5-HT1B/1D antagonist	Cellular regulation & physiology
Demethylasterriquinone B1	-0.15	0.53	-2.16	0.78	2.01	0.34	Selective insulin RTK activator	Cellular regulation & physiology
Tyrphostin B44, (+) enantiomer	-0.16	0.61	-2.16	0.47	2.00	0.23	EGFR-kinase inhibitor	Cellular regulation & physiology
PD 102807	-0.33	0.50	-2.29	0.37	1.97	0.18	Selective M4 antagonist	Neuromodulators
Ro 90-7501	-0.74	1.02	-2.69	1.21	1.95	0.54	Inhibitor of Aβ42 fibril formation	Cellular regulation & physiology
Aminopurvalanol A	-1.92	1.08	-3.66	0.48	1.74	0.30	Cyclin-dependent kinase inhibitor	Proliferation and differentiation
Gossypol	-0.62	1.16	-2.19	0.72	1.57	0.38	Proapoptotic; downregulates Bcl-2 and Bcl-XL	General Poison
PALDA	-0.91	0.92	-2.15	0.64	1.24	0.33	Endogenous lipid affecting TRPV1 receptors	Cellular regulation & physiology
OLDA	-1.97	0.45	-2.98	0.73	1.00	0.32	TRPV1 agonist	Cellular regulation & physiology

Supplementary Table 5: Source, HPV status and growth media of cell lines in the high-throughput study (HTS) and dose-response validation studies (DRVS)

Cell line	HPV status	Source	Study	Media
HMS-001	Positive	Rocco	HTS & DRVS	DMEM/F12
UM-SCC47	Positive	Carey	HTS & DRVS	DMEM/F12
93-VU-147T	Positive	deWinter	HTS & DRVS	DMEM/F12
UPCI:SCC090	Positive	Gollin	HTS & DRVS	DMEM/F12
UPCI:SCC154	Positive	Gollin	HTS & DRVS	DMEM/F12
UD-SCC2	Positive	Rocco	HTS & DRVS	DMEM/F12
UWO37	Positive	In House	DRVS	DMEM/F12
UWO23	Positive	In House	DRVS	DMEM/F12
SCC9	Negative	ATCC	HTS	DMEM/F12
FaDu	Negative	ATCC	HTS	DMEM/F12
Detroit 562	Negative	ATCC	HTS & DRVS	DMEM/F12
SCC15	Negative	ATCC	HTS & DRVS	DMEM/F12
SCC4	Negative	ATCC	HTS	DMEM/F12
SCC25	Negative	ATCC	HTS & DRVS	DMEM/F12
Cal 27	Negative	ATCC	HTS & DRVS	DMEM/F12
HSC2	Negative	JHSF	HTS & DRVS	DMEM/F12
JHU011	Negative	Rocco	HTS & DRVS	DMEM/F12
Cal33	Negative	DSMZ	HTS & DRVS	DMEM
JHU029	Negative	Rocco	HTS & DRVS	DMEM/F12
PCI 6B	Negative	Ferris	HTS & DRVS	DMEM/F12
PCI 13	Negative	Ferris	HTS	DMEM/F12
PCI 22B	Negative	Ferris	HTS	DMEM/F12
PCI 6A	Negative	Ferris	HTS	DMEM/F12
RF 15A	Negative	Ferris	HTS & DRVS	DMEM/F12
RF 15B	Negative	Ferris	HTS	DMEM/F12
RF 22A	Negative	Ferris	HTS	DMEM/F12
RF 37B	Negative	Ferris	HTS & DRVS	DMEM/F12
JHU 006	Negative	Rocco	HTS	DMEM/F12

Legend: **HPV** – human papillomavirus; **HTS** – High-throughput study; **DRVS** – Dose-response studies; **Rocco** - Dr. James Rocco, Harvard Medical School; **Carey** - Dr. Tom Carey, University of Michigan; **deWinter** - Dr. Johann deWinter, Free University of Amsterdam; **Gollin** - Dr. Suzanne Gollin, University of Pittsburgh Cancer Institute; **JHSF** - Japan Health Sciences Foundation; **DSMZ** - Deutsche Sammlung von Mikroorganismen und Zellkulturen; **Ferris** - Dr. Robert Ferris, University of Pittsburgh Cancer Institute; **In House;** Developed in our laboratory at Western University.

Supplementary Table 6: Short tandem repeat (STR) profiles of HNSCC cell line panel

Cell Line	Amelogenin	CSF1PO	D13S317	D16S539	D5S818	D7S820	TH01	TPOX	vWA	D18S51	D19S433	D21S11	D2S1338	D3S1358	D8S1179	FGA
93-VU-147T	X, Y	11, 11	8, 11	9, 12	11, 12	12, 12	6, 6	8, 8	17, 17	15, 20	12, 13	28, 29	21, 21	15, 15	12, 15	24, 24
UD-SCC2	X, Y	11, 12	8, 8	11, 13	10, 11	8, 9	8, 9	8, 10	18, 18	ND	ND	30, 31.2	ND	ND	ND	ND
HMS001	X, Y	11, 11	8, 8	9, 12	11, 12	12, 12	6, 6	8, 8	17, 17	15, 20	13, 14	28, 29	21, 21	15, 15	13, 15	24, 24
UM-SCC47	X, Y	11, 13	8, 11	8, 13	11, 12	11, 11	7, 9.3	10, 11	18, 18	18, 18	14, 15	29, 30	25, 25	15, 15	15, 15	25, 25
UPCI:SCC090	X, Y	11, 12	11, 11	12, 13	11, 12	9, 10	7, 7	8, 8	17, 17	14, 18	12, 13	39, 31	22, 22	14, 14	12, 12	20, 20
UPCI:SCC154	X, Y	10, 12	9, 12	13, 13	11, 12	9, 10	7, 7	8, 9	17, 17	15, 15	15.2, 16	28, 29	25, 25	16, 16	12, 12	20, 24
Cal27	X, X	10, 12	10, 11	11, 12	11, 12	10, 10	6, 9.3	8, 8	14, 17	13, 13	14, 15.2	28, 29	23, 24	16, 16	13, 15	25, 25
Detroit 562	X, X	11, 13	12, 12	11, 11	11, 12	8, 10	8, 9	8, 10	16, 16	15, 15	14, 14	28, 30	25, 25	15, 16	13, 13	21, 21
FaDu	----	12, 12	8, 9	11, 11	12, 12	11, 12	8, 8	11, 11	15, 17, 18	16, 16	14, 16	31.2, 31.2	19, 19	17, 18	13, 13	25, 25
SCC-4	X, Y	11, 11	11, 13	12, 12	13, 13	9, 11	9.3	8, 8	15, 17	15, 15	12, 14	32.2, 32.2	16, 24	18, 18	14, 14	21, 22
SCC-9	X, Y	11, 11	9, 9	10, 11	12, 12	8, 8	8, 9	9, 11	17, 17	12, 14	12, 14	28, 28	19, 21	15, 15	13, 13	20, 25
SCC-15	X, Y	10, 13	9, 14	12, 15	12, 12	10, 11	9, 9.3	8, 8	15, 17	16, 16	12, 15	30, 30	16, 23	16, 16	10, 13	24, 24
SCC-25	X, X	10, 10	13, 13	11, 12	12, 12	12, 12	8, 8	8, 12	17, 19	16, 16	13, 14	30, 30	17, 19	17, 17	13, 13	20, 24
Cal33	X, Y	11, 12	8, 13	11, 11	11, 12	8, 10	9, 9.3	8, 8	17, 17	14, 14	14, 15.2	29, 30	20, 25	17, 17	13, 13	21, 22
HSC2	X, Y	12, 13	11, 12	12, 12	10, 12	9, 12	6, 7	8, 8	16, 18	ND	ND	31.2, 31.2	ND	ND	ND	ND
JHU006	X, Y	10, 10	11, 11	12, 12	12, 12	9, 10	6, 6	8, 8	14, 16	16, 16	14, 14.2	31, 31	17, 25	13, 13	13, 14	19, 19
JHU011	X, X	10, 12	12, 12	9, 14	9, 12	11, 11	6, 9	8, 9	16, 17	13, 15	13, 14	31, 31	17, 26	18, 18	13, 13	23, 23
JHU029	X, Y	8, 12	12, 13	10, 13	13, 15	10, 11	8, 8	9, 11	15, 15	15, 15	13, 13	33.2, 33.2	19, 26	16, 16	14, 14	22, 25
PCI6A	X, X	12, 12	9, 11	11, 11	11, 12	8, 12	6, 6	11, 11	15, 18	12, 17	15, 16.2	31.2, 31.2	17, 24	18, 18	14, 14	23, 23
PCI6B	X, Y	10, 11	10, 11	12, 13	12, 12	12, 13	6, 7	8, 9	16, 18	16, 16	12, 14	32.2, 32.2	23, 24	18, 18	13, 14	20, 20
PCI13	X, X	10, 14	10, 11	11, 11	11, 14	11, 12	9, 9.3	6, 8	13, 17	16, 16	13, 15.2	29, 30	24, 24	14, 14	12, 13	22, 22
PCI30	X, Y	10, 10	13, 13	12, 13	11, 11	10, 12	6, 6	10, 11	14, 17	12, 15	12, 12	30, 32.2	18, 18	15, 15	13, 15	21, 21
RF15A	X, X	10, 12	12, 12	9, 9	10, 12	10, 11	6, 6	8, 11	17, 17	16, 18	13, 14	29, 29	20, 23	16, 16	14, 14	22, 22
RF15B	X, Y	11, 11	11, 11	11, 11	11, 11	11, 11	6, 6	8, 8	18, 18	14, 14	14, 15.2	28, 28	20, 25	16, 16	14, 14	23, 23
RF22A	X, X	10, 10	8, 12	9, 11	12, 12	8, 9	6, 6	8, 11	15, 18	18, 18	14, 15	28, 28	17, 20	16, 16	11, 13	22, 24
RF22B	X, X	10, 12	8, 11, 12	9, 12, 13	10, 12, 13	9-12	6, 6	8, 11	15-18	16, 18	12, 13, 14	27, 29	20, 22, 23	16, 18	13-16	18, 22
RF37A	X, X	11, 11	10, 13, 14	11, 14	11, 11	7, 10	7, 9.3	8, 11	16, 17	13, 13	15, 16	30, 32.2	24, 24	17, 17	11, 13	21, 21
RF37B	X, X	10, 12	12, 12	9, 9	10, 12	10, 11	6, 6	8, 11	17, 17	16, 18	13, 14	29, 29	20, 23	16, 16	14, 14	22, 22
SCC61	X, Y	10, 12	10, 12	9, 11	12, 13	8, 12	7, 9	8, 8	16, 17	ND	ND	28, 30	ND	ND	ND	ND
BICR56	X, X	12, 12	12, 12	11, 11	11, 12	8, 12	9, 9.3	8, 9	15, 16	14, 17	14, 15	28, 29	24, 26	14, 14	11, 13	21, 21
PE/CA-PJ49	X, X	11, 12	8, 11	11, 11	8, 12	8, 9	6, 10	8, 8	16, 19	24, 24	14, 14	32, 32	17, 18	16, 16	10, 11	20, 20

ND: not determined.
